# PRECONDITIONING OF PORCINE FLEXOR TENDONS FOR APPLICATION IN RECONSTRUCTION OF HAND FLEXOR TENDONS

**DOI:** 10.1590/1413-785220253306e295649

**Published:** 2025-11-10

**Authors:** Raquel Bernardelli Iamaguchi, Cesar Augusto Martins Pereira, Gustavo Bispo dos Santos, Flavio Elias Santiago do Nascimento, Heitor Pereira Vale da Costa, Rames Mattar

**Affiliations:** 1 Universidade de Sao Paulo, Faculdade de Medicina, Hospital das Clinicas, Grupo de Cirurgia da Mao e Microcirurgia Reconstrutiva, Sao Paulo, SP, Brazil.

**Keywords:** Active Mobility, Swine, Sutures, Tendons, Mobilidade Ativa, Suínos, Suturas, Tendões

## Abstract

**Objective:**

In chronic hand flexor tendon reconstruction with tendon grafts, the challenge is to obtain the best resistance and tension of the suture that allows early active mobility. This experimental study of tension relaxation aims to investigate whether prior preconditioning of the tendon graft could assist to identify the ideal tendon graft tension in these reconstructions.

**Methods:**

The porcine flexor tendons were subjected to the tension relaxation test, with three test cycles each with up to 50 N of tension and relaxation for 300 seconds. Measured: maximum force (N), maximum tension (Mpa) and maximum deformation.

**Results:**

After the peak tension of 50 N, the following was observed: maximum deformation, with an average tendon elongation of 2.3 mm; average residual tendon elongation of 0.6 mm; demonstrating the viscoelastic spring characteristic of porcine tendons.

**Conclusion:**

We recommend performing intraoperative preconditioning of the tendon graft with loads close to active grip strength (50 N to 70 N). If it is impossible to perform preconditioning, the suture can be placed 17 degrees of flexion of the proximal interphalangeal joint above the cascade flexion of fingers, compensating for tendon elongation under a load of 50 N. ***Level of Evidence III; Experimental***.

## RESUMO

**Objetivo:**

Na reconstrução de lesão crônica de tendão flexor da mão, o desafio é a obtenção da melhor resistência e tensão da sutura que possibilite a mobilidade ativa precoce. Este estudo experimental de relaxamento a tensão, tem como objetivo investigar se o pré- condicionamento prévio do enxerto de tendão poderia auxiliar na identificação da tensão ideal do enxerto de tendão nestas reconstruções.

**Métodos:**

Os tendões flexores suínos foram submetidos ao ensaio de relaxamento à tensão, com três ciclos de testes cada até 50N de tensão e relaxamento por 300 segundos. Mensurados: a força máxima (N), a tensão máxima (Mpa) e a deformação máxima.

**Resultados:**

Após o pico de tensão de 50 N, foi observado: deformação máxima, com alongamento do tendão médio de 2,3 mm; alongamento médio residual de 0,6 mm; demonstrando a característica viscoelástica de mola dos tendões suínos.

**Conclusão:**

Recomendamos a realização do pré-condicionamento intraoperatório do enxerto de tendão flexor com cargas próximas à força de preensão ativa (50N a 70N). Na impossibilidade da realização do pré-condicionamento, a sutura pode ser realizada com 17 graus de flexão da interfalângica proximal acima da flexão em cascata dos dedos, compensando o alongamento do tendão sob carga de 50 N. ***Nível de Evidência lll; Experimental***.

## INTRODUCTION

Chronic flexor tendon injury of the hand represents a significant reconstructive challenge for hand surgeons. Among the therapeutic options are tendon transfers, arthrodesis, and tendon grafting. In cases of severe mobility limitation and complex lesions, amputation of nonfunctional fingers may be indicated. Flexor tendon reconstruction with tendon grafts^1^ is the most common option, but it faces technical challenges such as graft adhesion, inadequate tension, and failure to restore range of motion.

To reduce postoperative complications, early active mobilization is advocated, aiming to promote tendon gliding and reduce adhesions and motion limitations. However, no objective data are available regarding the optimal surgical tension for tendon grafts. Studies on stress-relaxation in autologous tendon grafts for knee ligament reconstruction^2^ suggest that graft preconditioning with 50 Newtons (N)^3,4^ may prevent postoperative laxity.

The present study aims to evaluate the viscoelastic properties of porcine flexor tendons through stress-relaxation testing. The objective is to determine the applicability of these properties in flexor tendon reconstruction using tendon grafts, in order to prevent elongation and laxity, thereby reducing common postoperative complications in flexor tendon reconstruction of the hand, such as increased flexion force due to inadequate graft tension.

## MATERIALS AND METHODS

Twenty-one stress-relaxation tests were performed on seven flexor tendons harvested from porcine carcasses used in the surgical technique program of our University. The tendons were collected from the hind limbs of pigs after approval by the institutional animal ethics committee (CEUA No. 1560/2020). The animals were euthanized with intraperitoneal thiopental sodium at a dose of 75 mg/kg, in accordance with the guidelines of the Brazilian College of Animal Experimentation (COBEA, 2007). After use in the surgical technique program, the carcasses designated for disposal were repurposed for this project. Both flexor tendons of the pigs’ hind limbs were collected after euthanasia.

Preliminary measurements of the porcine flexor tendon diameter were obtained using a caliper (mm). Three measurements were taken, and their mean was calculated to minimize error, from which the total cross-sectional area (mm²) was determined.

The tendons were immediately prepared for testing. Both ends of the tendons were positioned in rectangular trapezoidal-profile clamps, previously designed to accommodate the average diameter of porcine flexor tendons.

The tendons were subjected to stress-relaxation testing using a Kratos mechanical testing machine, model 5002, equipped with a 981 N (100 kgf) load cell adjusted to a 98.1 N (10 kgf) scale. A Lynx data acquisition system, model ADS2000, recorded force and displacement data from the machine at a rate of 10 samples per second and transferred them to a personal computer via software that enabled visualization and recording of the acquired data over time (resolution of 100.0 milliseconds).

**Figure 1:**
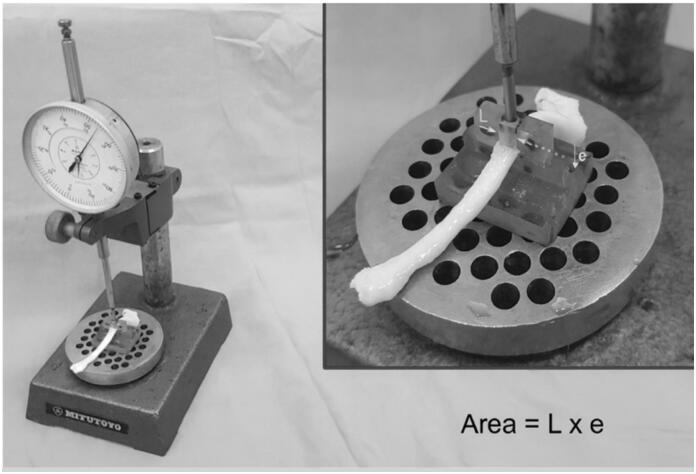
The area is calculated by multiplying the actuator width (L = 4.7 mm) by the thickness (e) measured with the dial indicator.

Source: Authors.

Before tendon fixation to the testing machine, each specimen was measured at three cross-sectional regions: at the tendon midpoint and 20 mm proximally and distally. Measurements were performed using a device consisting of a dial indicator (Mitutoyo, resolution 0.01 mm), a channel measuring 4.7 mm in width and 8 mm in depth, and a parallelepiped-shaped actuator sliding along the channel, coupled to the dial indicator stem. The cross-sectional area was calculated by multiplying the channel width of 4.7 mm by the thickness measured by the dial indicator. (Figure 1)

Two rectangular clamps with a sinusoidal profile (6.5 mm pitch) were used. Each clamp had four screws with lock washers to ensure that the clamping force on the tendon remained less variable during the test. One clamp was fixed to the base of the testing machine using a bench vise, and the other clamp was attached to the load cell (mobile part) using a universal joint. The tendon ends were secured in the clamps so that the central portion to be tested was set at a distance of 35 mm between clamps.

With the specimen positioned in the testing machine, a load of 50 N was applied for 10 seconds. After this procedure, the load was released and the clamp screws were retightened. The tendon was rehydrated with 0.9% sodium chloride solution for 20 minutes to ensure recovery of its initial length. Then, the movable clamp was repositioned until the load returned to zero, and the distance between the clamps, corresponding to the initial tendon length, was measured using a Mitutoyo caliper with a resolution of 0.05 mm.

The test consisted of pulling the tendon at a speed of 5 mm/min until reaching a load of 50 N, at which point the testing machine immediately stopped its upward motion, producing elongation of the material within its elastic zone. After 300 seconds, the machine reversed its motion, returning to the point where the recorded load was equal to zero, at which point it immediately stopped again. After an additional 300 seconds, the test was completed. Throughout the procedure, the tendon was hydrated with 0.9% sodium chloride solution.

**Figure 2:**
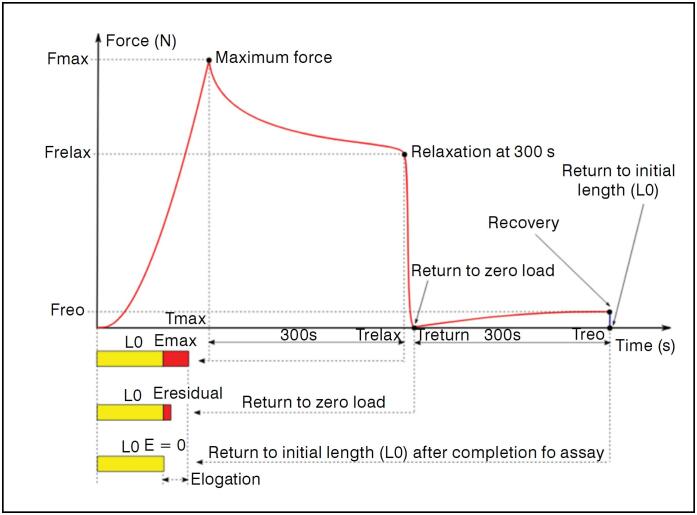
Hypothetical graph of force as a function of time during a stress-relaxation test, highlighting the phases of 50 N load application (Peak), stress relaxation after 300 s, tendon return to zero load, and recovery after 300 seconds. Where: Fpico and Tpico: force and time at peak; Frelax and Trelax: force and time at 300 s relaxation; Tretorno: time at the moment of return to zero load; Frec and Trec: force and time after 300 s of recovery; L_0_: initial tendon length; Dpico: deformation at the load peak; Dresidual: deformation after return to zero load.

Due to the viscoelastic behavior of the material, during the first 300 seconds there was a loss of tension evidenced by the decrease in load, and in the final 300 seconds a slight increase in load was recorded, corresponding to the material's attempt to recover its initial length. At the end of the test, the machine recorded a load and displacement value, which was reset once the crosshead returned to the tendon's initial length. Figure 2 illustrates a hypothetical stress-relaxation curve of the tendon test, showing the main phases of the procedure: peak, relaxation at 300 s, return to zero load, and recovery.

Each tendon underwent three repetitions of stress-relaxation tests with a 50 N load, always maintaining a 30-minute interval between repetitions.

The calculated parameters were relaxation force (Frelax) and stress (srelax), relative deformation at peak (ε%pico), absolute residual deformation (Dres) relative residual deformation (D%res), and recovery force (Frec) and stress (srec).

Stresses were calculated as the ratio of force (N) to the mean cross-sectional area (mm²) of the three tendon regions, expressed in MPa. Relative deformation at peak (ε%pico) was calculated as the ratio of absolute residual deformation (Dres) to the initial tendon length, multiplied by 100.

At the moment of peak stress of the flexor tendon, four measurements were performed:

1. Maximum force reached (N)
2. Maximum stress (MPa)
3. Maximum tendon deformation (mm)
4. Percentage deformation, based on comparison with the mean of the initial unstressed measurements (mm)

Following the stress-relaxation test, the four measurements described at the 50 N peak load were repeated at the following time points:

1. Tendon relaxation after 300 seconds (5 minutes) from peak stress
2. Return to zero load (N)
3. Characteristics after return, including residual tendon deformation

A descriptive study was conducted using SPSS. Statistical analysis was performed with SPSS software version 20.0 (SPSS Inc®, Chicago, IL, USA), employing descriptive statistics and inferential statistical analysis.

## RESULTS

The mean cross-sectional area of the porcine tendons used in the stress-relaxation tests was 11.4 mm² (standard deviation [SD] 2.7), with a mean diameter of 29.7 mm (SD 2.5 mm). The mean tendon length, calculated from the average of three different measurements, was 36.5 mm (SD 2.6).

During the peak stress of the test, the force achieved was 49.3 N (SD 0.80 N), with the target load of approximately 50 N reached in the trial. At this moment, the peak stress averaged 4.7 MPa (SD 1.3 MPa); peak deformation with tendon elongation averaged 2.3 mm (SD 0.4 mm), with a deformation percentage—based on the mean of initial unstressed measurements—of 6.2% (SD 0.9%); and peak stiffness of the porcine tendon graft was 57.5 N/mm (SD 15.0 N/mm).

After the 50 N peak load, the following values were obtained after relaxation to zero load in Newtons for the porcine flexor tendon, compiled from all test cycles: the mean time to return to zero stress was 360.8 seconds (SD 10.3 seconds); mean residual elongation was 0.6 mm (SD 0.18 mm); and the mean residual deformation percentage was 1.6% (SD 0.5).

The values for peak deformation and residual deformation were described separately for the three different cycles of deformation and stress-relaxation of each porcine flexor tendon. (Table 1)

**Table 1:** General values and after each 50 N tensioning cycle (first, second, and third cycles).

	Mean	SD	Minimum	Maximum
Dpico	2.26	0.38	1.73	2.90
Dresidual	0.59	0.18	1.73	2.91
Dpico (1.o cicle)	2.31	0.42	1.75	2.90
Dresidual (1.o cicle)	0.54	0.17	0.30	0.77
Dpico (2.o cicle)	2.27	0.43	1.76	2.75
Dresidual (2.o cicle)	0.69	0.19	0.42	1.01
Dpico (3.o cicle)	2.31	0.42	1.75	2.90
Dresidual (3.o cicle)	0.54	0.17	0.30	0.77

Legend: Deformation in mm at the 50 N peak load (Dpico); residual deformation after return to zero load (Dresidual); standard deviation (SD).

After statistical evaluation, no statistically significant differences were observed among the three different cycles regarding the mean deformation data at the 50 N peak load (p = 0.19) and residual deformation after return to zero load, as assessed by ANOVA in SPSS (p = 0.14).

## DISCUSSION

The tension of the tendon graft for reconstruction of chronic flexor tendon injuries is empirically determined, with reconstruction usually performed for the flexor digitorum profundus.^5^

Previous experimental studies have demonstrated that porcine flexor tendons are compatible with human flexor tendons and are suitable for comparative experimental models, behaving similarly to tendon grafts.^6^ The most common human flexor tendon graft sources are the palmaris longus, plantaris, flexor digitorum superficialis, semitendinosus, or gracilis tendons^6^ and more recently, allografts.^7^

According to biomechanical evaluations of hand flexor tendons,^8^ we know that for finger flexion with the wrist in neutral position, there is a total displacement of 32 mm (15–43 mm) of the flexor digitorum profundus during its full excursion.^9^ The following tensions are observed:

- Passive flexion: 2–4 N of force
- Active (light) flexion: 10 N of force
- Active (strong) flexion: 50–70 N / pinch: 120 N of force

Preconditioning of porcine flexor tendons at 50 N is based on experimental preconditioning studies of grafts for anterior cruciate ligament reconstruction^2-4^ and also on the load corresponding to strong active flexion. The biomechanical properties of the flexor tendon^10^ should not be analyzed linearly, but rather in terms of the tendon's viscoelasticity after stress and relaxation, which are time- and load-dependent, leading to constant elongation and relaxation. Previous studies, such as that by Monleon,^11^ observed that under hydration conditions and after two preconditioning cycles, the viscoelastic characteristics of human flexor tendons resemble spring-like behavior. However, it is important to note that tendon behavior may change with repeated loading, as the tissue absorbs load and energy after each cycle.^10^

In the context of flexor tendon reconstruction of the hand, the key question is: what would be the ideal behavior of an avascular tendon graft to simulate the optimal tension of a flexor digitorum profundus tendon?

In this experimental study, for tendon preconditioning we applied a 5 kg (50 N) load. After stress-relaxation, the residual deformation was 1.6% of the initial length. With these preconditioned tendons, the return to zero load and zero deformation took an average of 6 minutes. This residual deformation of 0.6 mm on average cannot be considered plastic deformation, since it may occur in experimental testing due to adverse technical conditions such as dehydration and/or failure of the machine clamps to properly hold the graft. Therefore, porcine tendons ultimately behave like a spring.

During surgery, this spring-like viscoelastic behavior allows the tendon graft to be sutured for flexor tendon reconstruction under optimal tension. With the advent and increasing use of the WALANT technique (Wide Awake Local Anesthesia No Tourniquet),^12^ the ideal graft tension can be determined intraoperatively, since elongation of the tendon graft can be directly observed and tested.^13^ In cases of tendon elongation during active flexion, there may be loss of strength and full excursion; conversely, excessive tension may cause a quadriga effect, leading to loss of flexion strength in adjacent fingers.^14,15^

In porcine flexor tendon grafts, under strong active flexion loads, with the 50 N peak used in this study, a maximum mean deformation of 2.3 mm was observed across the three preconditioning cycles. Upon return to zero load, the tendons behaved like a spring, with insignificant residual deformation (1.3% of tendon length), likely explained by graft dehydration during experimental testing. Considering biomechanical studies describing ∼1.3 mm of tendon excursion per 10° of joint rotation,^16,17^ and comparing with our mean peak deformation values at 50 N, postoperative deformation in flexor tendon reconstruction without preconditioning could lead to elongation under an active 50 N load, altering reconstructed tendon function by up to 17° of proximal interphalangeal joint rotation—solely due to tendon deformation, without even considering the risk of suture site laxity.

Therefore, we recommend surgical reconstruction of chronic or irreparable flexor digitorum profundus tendon injuries with tendon grafting under local anesthesia. The surgical technique should begin with distal graft fixation at the distal phalanx or the remnant flexor digitorum profundus stump, followed by intraoperative graft tensioning through cyclic and active finger flexion movements performed by the patient. After proper tensioning, proximal graft fixation should be completed, with additional active flexion tests to confirm adequate graft tension and detect possible elongation. As a second option, if WALANT surgery is not feasible, we suggest intraoperative preconditioning of the flexor tendon graft with loads close to active grip strength (50–70 N). As a third alternative, if neither option is possible, graft suturing can be performed with a shortening of approximately 2.3 mm (mean peak deformation value in our study) or with an additional 17° of proximal interphalangeal joint flexion beyond the normal flexion cascade of the finger (based on prior reports of 1.3 mm tendon excursion for every 10° loss of flexion).^16^

Study limitations include: preconditioning was limited to three linear cycles (a small number, but chosen to avoid graft degradation since this was an in vivo study using porcine tendon grafts); the sample size; and the fact that the actual conditions of tendon graft reconstruction in humans may not be comparable to our experimental study on porcine flexor tendons using the Kratos® universal testing machine.

## CONCLUSION

We recommend intraoperative preconditioning of the flexor tendon graft with loads close to active grip strength (50–70 N). If preconditioning is not feasible, the suture may be performed with 17 degrees of proximal interphalangeal joint flexion beyond the natural flexion cascade of the fingers, thereby compensating for tendon elongation under a 50 N load.
